# Efficient double-flowered gentian plant production using the CRISPR/Cas9 system

**DOI:** 10.5511/plantbiotechnology.23.0424a

**Published:** 2023-09-25

**Authors:** Masahiro Nishihara, Akiko Hirabuchi, Fumina Goto, Aiko Watanabe, Chiharu Yoshida, Rie Washiashi, Masashi Odashima, Keiichirou Nemoto

**Affiliations:** 1 Iwate Biotechnology Research Center, 22-174-4 Narita, Kitakami, Iwate 024-0003, Japan; 2 Iwate Agricultural Research Center, 20-1 Narita, Kitakami, Iwate 024-0003, Japan

**Keywords:** CRISPR/Cas9, double flower, genome editing, gentian, null segregant

## Abstract

Japanese cultivated gentians are highly valued ornamental flowers in Japan, but the flower shape is mostly limited to the single-flower type, unlike other flowers such as roses and carnations. To overcome this limitation, we used the CRISPR/Cas9 genome editing system to increase double-flowered genetic resources in gentians. Our approach targeted an *AGAMOUS* (*AG*) floral homeotic gene (*AG1*), which is responsible for the natural mutation that causes double flowers in gentians. We designed two targets in exon 1 of *AG1* for genome editing and found that 9 of 12 herbicide-resistant shoots had biallelic mutations in the target regions of *AG1*. These nine lines all produced double flowers, with stamens converted into petaloid organs, similar to the natural mutant. We also analyzed the off-target effects of *AG2*, which is homologous to *AG1*, and found that such effects occurred in gentian genome editing but with low frequency. Furthermore, we successfully produced transgene-free genome-edited plants (null segregants) by crossing with wild-type pollen. F_1_ seedlings were subjected to PCR analysis to determine whether foreign DNA sequences, two partial regions of the CaMV35S promoter and *Cas9* gene, were present in the genome. As a result, foreign genes were segregated at a 1 : 1 ratio, indicating successful null segregant production. Using PCR analysis, we confirmed that four representative null segregants did not contain transfer DNA. In summary, our study demonstrates that the CRISPR/Cas9 system can efficiently produce double-flowered gentians, and null segregants can also be obtained. These genome-edited plants are valuable genetic resources for future gentian breeding programs.

## Introduction

Most ornamental flowers have been bred to exhibit double flowers, with many petals arranged in multiple layers, because double flowers are visually appealing and have great consumer appeal (Ferguson 2018). Double-flowered cultivars have been commercialized for major ornamental flowers such as roses, carnations, and lilies as well as other flowers like lisianthus, petunia, stock, and cyclamen, among others. Conventional breeding techniques using natural or induced mutants have been used to develop these cultivars. In Japanese cultivated gentians, DNA marker-assisted selection has been used to produce double-flowered cultivars from a natural double-flowered mutant (Tasaki et al. 2017). Four double-flowered cultivars bred in Iwate Prefecture and Hachimantai City are now sold mainly for gift-giving purposes. However, the number of double-flowered gentian cultivars is still limited, and further development is necessary to meet consumer demand and increase sales.

Currently, there is only one available genetic resource for the double-flowered trait in gentians. The mutation of the *AGAMOUS* (*AG)* gene *GsAG1*, a well-known homeotic gene that belongs to the C-class MADS box gene family, caused by the insertion of the LTR-retrotransposon (*Tgs1*) in the sixth intron, is responsible for the double-flowered phenotype (Nakatsuka et al. 2015). To efficiently develop more double-flowered gentian cultivars, novel alleles or direct mutation of *AG1* in existing cultivars are promising approaches, as the original double-flowered mutant has several undesirable traits as a breeding material. These include many green spots in the corolla (Takahashi et al. 2020), narrow leaves compared with those of elite cultivars, and late flowering time. Several rounds of backcrossing are required to develop novel cultivars, and Japanese cultivated gentians are perennial plants, so the process is time-consuming and arduous. Ion beam irradiation has been used for mutagenesis in gentians (Sasaki et al. 2018), but it is impossible to achieve targeted mutagenesis using this method as the mutations occur randomly.

Genome editing technology offers a promising solution to the challenges faced in developing double-flowered gentian cultivars. In particular, a CRISPR/Cas9-mediated genome editing system has been developed for gentians and used to produce mutants with various agronomic traits. For example, flower color has been altered by targeting three anthocyanin modification genes encoding anthocyanin 5-*O*-glucosyltransferase, anthocyanin 3′-*O*-glucosyltransferase, and anthocyanin 5/3′-aromatic acyltransferase (Tasaki et al. 2019). The glutathione *S*-transferase gene, *GST1*, which is involved in anthocyanin transport, was also identified by genome editing technology (Tasaki et al. 2020). In 2022, we generated genome-edited lines of *FT2* and *FRUITFULL* and confirmed their roles in the phase transition of overwintering buds (Takahashi H et al. 2022). We also extended flower longevity by knocking out the NAC transcription factor (*EPH1L*) involved in flower senescence in gentians (Takahashi S et al. 2022). However, to the best of our knowledge, there are no reports on modifying flower shape through genome editing in Japanese cultivated gentians.

Double flowers in many ornamental plants are often derived from mutations in floral homeotic MADS box genes. *AGAMOUS* is responsible for stamen and carpel formation in the third and fourth whorls of wild-type (WT) flowers. Changes in *AGAMOUS* expression play an essential role in double flower formation in roses (Dubois et al. 2010), carnations (Jin et al. 2022), camellias (Sun et al. 2014), cyclamens (Mizunoe et al. 2015), *Thalictrum thalictroides* (Galimba et al. 2012), magnolias (Zhang et al. 2015), evergreen azaleas (Cheon et al. 2017), *Kerria japonica* (Ma et al. 2018), and *Tricyrtis macranthopsis* (Sharifi et al. 2015). Although not all mutations in *AGAMOUS* and *AGAMOUS*-like genes are associated with double-flowered plants, the loss of function of C-class genes is likely to be involved in the ornamental double-flowered phenotype. RNAi-mediated silencing of C-class MADS box genes belonging to the AG-clade has induced double flower formation in petunias (Noor et al. 2014), and a chimeric repressor gene silencing *AG* homolog in morning glories also resulted in the same phenotype (Sage-Ono et al. 2011). In addition, B-class MADS box genes are thought to be involved in the petaloidy of stamens in cultivated amaryllis (Li et al. 2022) and double flower development in camellias (Gaopu 2011). Furthermore, an *APETALA2* homolog (A-class gene) belonging to the AP2/ERF transcription factor family regulates the number of rose petals in response to temperature fluctuation (Han et al. 2018). *AP2* homolog genes are also involved in double flower formation in carnations, petunias, peaches and roses (Gattolin et al. 2020, 2018; Wang et al. 2020). Balancing the expression of these homeotic genes among whorls is essential for determining floral organ identity and ultimately flower shape.

Japanese cultivated gentians (*Gentiana triflora*, *Gentiana scabra*, and their hybrids), which belong to the Gentianaceae family, are commonly used as cut flowers and potted plants (Nishihara et al. 2018). Previous studies have identified 14 MADS box genes belonging to A–E classes in *G. scabra* (Nakatsuka et al. 2016, 2015). The double-flowered phenotype has been attributed to decreased expression of *GsAG1* due to the insertion of a retrotransposable element (*Tgs1*) into the sixth intron, as demonstrated by molecular dissection of a mutant line, and silencing of the *GsAG1* gene via ALSV-mediated gene silencing resulted in double flowers (Nakatsuka et al. 2015). However, to date, genome editing of the gentian *AG1* gene for flower shape modification has not been reported in gentians.

In this study, we aimed to use the CRISPR/Cas9-mediated genome editing system to produce double flowers in gentians. To achieve this, we generated and analyzed genome-edited lines of gentian *AG1*. Additionally, we produced null segregants through crossing and analyzed them. Our results suggest that these *AG1*-edited gentians can serve as valuable genetic resources for gentian breeding, facilitating an increase in *ag1* alleles for double flower production.

## Materials and methods

### Plant materials

In this study, we used the hybrid gentian ‘Albireo’ (*G. scabra×G. triflora*), which has been maintained in vitro for over 20 years. The cultivar was cultured on half strength MS medium supplemented with 3% (w/v) sucrose and 0.2% (w/v) gellan gum and grown at 20°C under a 16/8 h light/dark cycle at a light intensity of approximately 30 µmol m^−2^ s^−1^.

### Vector construction and *Agrobacterium*-mediated transformation

**Figure 1:**
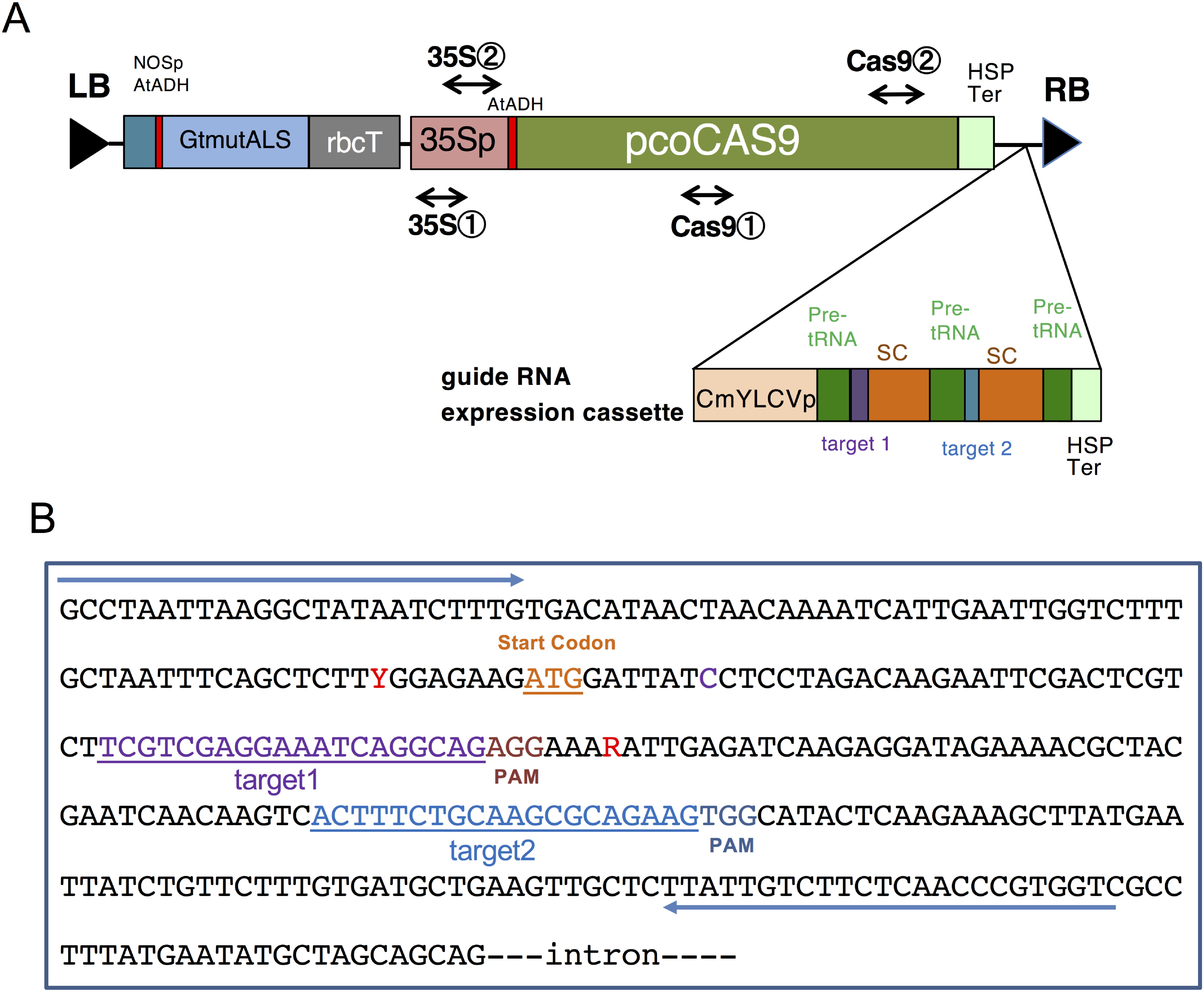
Figure 1. Schematic diagram of the binary vector and target sequence in the gentian *AG1* gene. (A) Map of the T-DNA region of pSALS-AG1. LB, left border of T-DNA; NOSp, nopaline synthase promoter; AtADH, 5′-untranslated region of the alcohol dehydrogenase gene of *Arabidopsis*; GtmutALS, gentian mutated acetolactate synthase; rbcT, *Arabidopsis* rubisco small subunit terminator; 35Sp, Cauliflower mosaic virus 35S promoter; pcoCas9, plant codon-optimized *Cas9* of *Streptococcus pyogenes*; Pre-tRNA, tRNA precursors; SC; single-guide RNA scaffold; HSPter, *Arabidopsis* heat shock protein 18.2 terminator; CmYLCVp, Cestrum yellow leaf curling virus promoter; RB, right border of T-DNA. (B) First exon sequence of *AG1* containing target sites 1 and 2 for genome editing. R and Y marked using IUPAC notation indicate SNPs between two alleles. PAM indicates the protospacer-adjacent motif. Arrows indicate the primers used for PCR amplification.

A binary vector, pSALS-AG1, was constructed (Figure 1A) to contain a mutated acetolactate synthase (*ALS*) gene of gentians for selection by an ALS-inhibiting herbicide, bispyribac-sodium (BS) (Figure 1A), and codon-optimized *Cas9* (Fauser et al. 2014). Guide RNAs were driven under the control of the promoter of Cestrum yellow leaf curling virus (CmYLCV) (Sahoo et al. 2014) and the terminator of *Arabidopsis* heat shock protein 18.2 (Nagaya et al. 2010). A transfer RNA processing system (Xie et al. 2015) was used to express two guide RNAs that targeted exon 1 of gentian *AG1* (Figure 1B). The binary vector was transformed into *Agrobacterium tumefaciens* EHA101 through electroporation and used for gentian transformation. Transgenic gentian plants were produced as described previously (Takahashi S et al. 2022).

### Confirmation of genome editing via sequence analysis

Sanger sequencing analysis was used to confirm genome editing in BS herbicide-resistant shoots. Crude DNA was extracted from small leaf sections crushed using toothpicks and subjected to PCR amplification using KOD One™ PCR Master Mix (Toyobo, Osaka, Japan) according to the manufacturer’s instructions. Following treatment with illustra™ ExoStar™ (GE Healthcare, Buckinghamshire, UK) to remove unincorporated primers, the products were sequenced using a BigDye Terminator ver. 1.1 Cycle Sequencing Kit (Applied Biosystems, Foster City, CA, USA) and an ABI PRISM 3500 Genetic Analyzer or SeqStudio Genetic Analyzer (Applied Biosystems). Lines exhibiting sequencing chromatograms that differed from the untransformed WT were subjected to further analysis. Amplicons of the screened putative genome-edited lines were subcloned into a pCR™Blunt II-TOPO® vector (Thermo Fisher Scientific, Waltham, MA, USA) and sequenced as described above. The primers used are listed in Supplementary Table S1.

### Flower phenotype of genome-edited gentian plants

Nine plants with biallelic mutations in *AG1* were selected and acclimatized for observation of flower shape. These plants were grown in a closed greenhouse at Iwate Biotechnology Research Center under natural daylight from spring to autumn until flowering. As a control, untransformed ‘Albireo’ was also grown under similar conditions.

### Off-target analysis

An *AG2* gene, a C-class MADS box gene homologous to *AG1*, is found in Japanese cultivated gentians (Nakatsuka et al. 2015). Therefore, to analyze *AG2*, Sanger sequencing was conducted and PCR analysis was performed in nine genome-edited lines to amplify *AG2* in the corresponding region of *AG1.* After subcloning, the sequences were determined using Sanger sequencing analysis as described above. The primers used are listed in Supplementary Table S1.

### Production of null segregants through crossing with WT pollen

The obtained genome-edited lines were double-flowered and did not produce pollen; therefore, two representative genome-edited lines, no. 1 and no. 14, were crossed with WT pollen (Iwate Agricultural Research Center, breeding lines: unk, HO, and MN). To promote growth, ovule culture was conducted as described previously (Takamura et al. 2019). PCR analysis was performed using crude DNA extracted from part of the seedlings to amplify two regions of the CaMV35S promoter and *Cas9* gene, respectively (Figure 1A). Subsequently, genomic DNA was extracted from approximately 100 mg of leaves of each two F_1_ individuals and primary genome-edited lines, no. 1 and no. 14, using a NucleoSpin™ Plant II Kit (Macherey-Nagel, Germany). They were analyzed using primer pairs to cover the whole transfer DNA (T-DNA) region, as shown in Supplementary Table S1 and Supplementary Figure S6. PCR was performed using 1 ng of genomic DNA or 1 pg of plasmid DNA, pSALS-AG1 by KOD One™ PCR Master Mix. The PCR conditions were as follows: 35 cycles of denaturation at 98°C for 10 s, annealing at 60°C for 5 s, and extension at 68°C for 5 s. PCR products were separated on a 1.6% agarose gel and stained with ethidium bromide.

## Results

**Figure 2:**
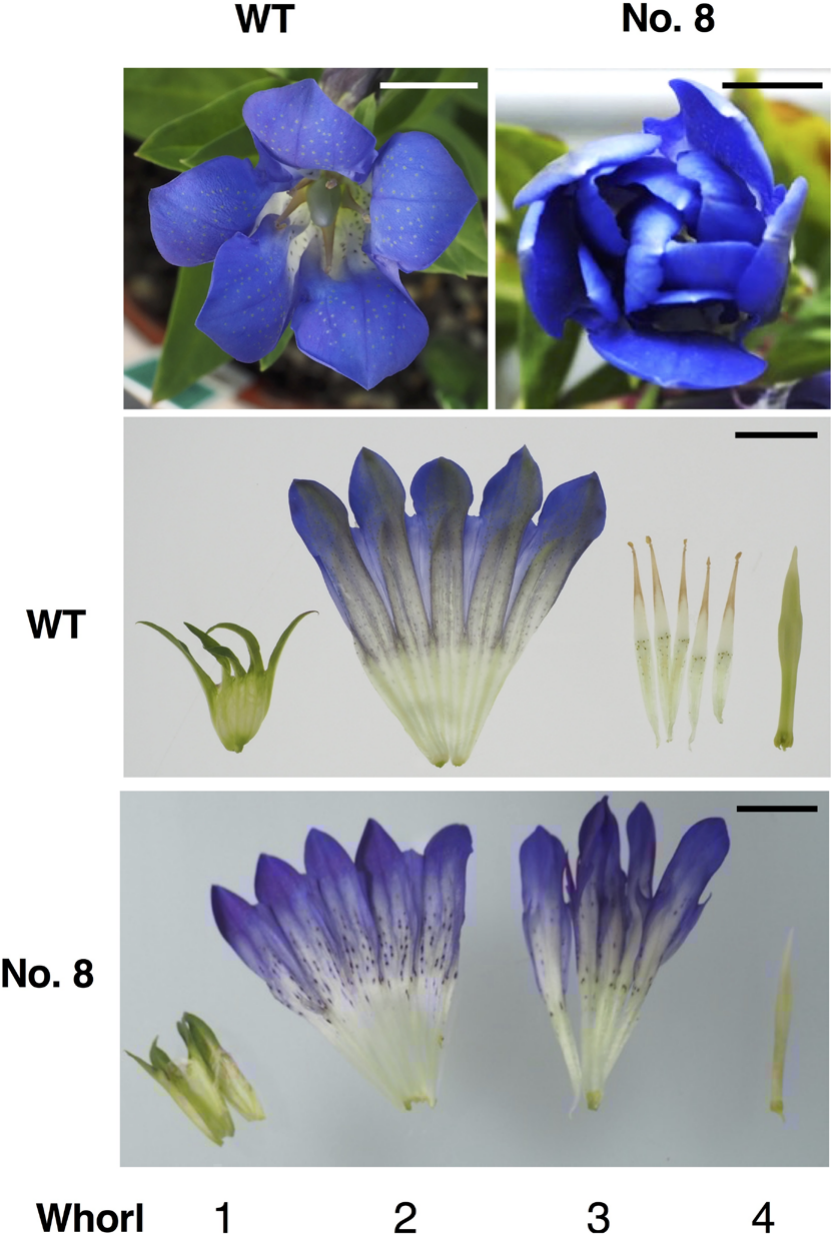
Figure 2. Typical flowers of *AG1* genome-edited gentians. Overhead view and dissected flowers of line no. 8 and WT ‘Albireo.’ In the WT, whorls 1, 2, 3, and 4 correspond to the sepal, petal, stamen, and carpel, respectively. In line no. 8, the conversion from stamen to petal in whorl 3 is shown. Scale bar: 1 cm.

Leaf sections of gentian ‘Albireo’ were inoculated with *A. tumefaciens* EHA101 harboring pSALS-AG1. Twelve BS-resistant calli were selected on shoot selection media, and BS-resistant shoots were obtained. Using crude DNA extracts from small parts of these shoots, PCR analysis was employed to amplify the *AG1* region containing targets 1 and 2, which produced products of the expected size (287 bp; Supplementary Figure S1). Extra longer bands were also amplified in lines no. 4, 5, and 8. Direct sequencing analysis of the PCR products revealed different sequencing chromatograms in 10 lines relative to those of the WT (Supplementary Figure S2). Among these lines, no. 3 had a WT sequence in one allele and no. 5 was similar to no. 4; therefore, the remaining eight lines were subcloned, and the sequences were determined. Table 1 summarizes the results, showing that all eight lines exhibited genome editing in target 1 and/or target 2, such as small in/del (from −18 bp to +1 bp). Larger insertions, such as +154 bp and +51 bp, were observed in lines no. 4 and 8, respectively. Line no. 12 showed two sequences on allele 1 and was considered a chimeric genome-edited line. All edited sequences led to premature stop codons and did not encode functional AG1 protein. In total, nine biallelic lines were acclimatized and cultivated until flowering in a closed greenhouse. As a result, all nine lines produced double flowers (Figure 2, Supplementary Figure S3), indicating a conversion from stamens to petals, whereas the WT ‘Albireo’ produced only single flowers.

**Table 1:** Table 1. *AG1* target sequences determined by Sanger sequencing analysis.

Line	Amplified band	Allele	Target 1	In/del	Target 2	In/del
WT	~300 bp	1	TCGTCGAGGAAATCAGGCAGAGG	WT	ACTTTCTGCAAGCGCAGAAGTGG	WT
		2	TCGTCGAGGAAATCAGGCAGAGG	WT	ACTTTCTGCAAGCGCAGAAGTGG	WT
No. 1	~300 bp	1	TCGTCGAGGAAATCAGGGCAGAGG	+1 bp	ACTTTCTGCA-------AAGTGG	−7 bp
		2	TCGTCGAGGAAATCAGGACAGAGG	+1 bp	ACTTTCTGCAAG-----AAGTGG	−5 bp
No. 4	~300 bp,	1	TCGTCGAGGAAATCAGGGCAGAGG	+1 bp	ACTTTCTGCAA----AGAAGTGG	−4 bp
	~450 bp	2	TCGTCGAGGAAATCAGGACAGAGG	+1 bp	ACTTTCTGCAAGCG(−12+166)	+154 bp
No. 5	Same pattern to No. 4	1	Not determined		Not determined	
		2	Not determined		Not determined	
No. 6	~300 bp	1	TCGTCGAGGAAATCAGGCAGAGG	WT	ACTTTCTGCAAG-----AAGTGG	−5 bp
		2	TCGTCGAGGAAATCA--CAGAGG	−2 bp	ACTTTCTGCAAGCGC-GAAGTGG	−1 bp
No. 8	~300 bp,	1	TCGTCGAGGAAA---------GG	−9 bp	ACTTTCTGC--------AAGTGG	−8 bp
	~350 bp	2	TCGTCGAGGAAATCAGG-AGAGG	−1 bp	A(+51)	+51 bp
No. 9	~300 bp	1	TCGTCGAGGAAATCAGGCAGAGG	WT	ACTTTCTGCAAGCGC--AAGTGG	−2 bp
		2	TCGTCGAGGAAATCAGGCAGAGG	WT	ACTTTCTGC--------AAGTGG	−8 bp
No. 12	~300 bp	1	TCGTCGAGGAAATCAGGCAGAGG	WT	AC------------------TGG	−18 bp
		1	TCGTCGAGGAAATCAGGCAGAGG	WT	AC---------------------	−23 bp
		2	TCGTCGAGGAAATCAGGACAGAGG	+1 bp	ACTTTCTGC--------AAGTGG	−8 bp
No. 13	~300 bp	1	TCGTCGAGGAAATCAGGACAGAGG	+1 bp	ACTTTCT----------AAGTGG	−10 bp
		2	TCGTCGAGGAAATCAGGTCAGAGG	+1 bp	ACTT----------------TGG	−16 bp
No. 14	~300 bp	1	TCGTCGAGGAAATCAGGGCAGAGG	+1 bp	ACTTTCTGCAAGCGC--AAGTGG	−2 bp
		2	TCGTCGAGGAAATCA--CAGAGG	−2 bp	ACTTTCTGCAAGCGCAGGAAGTGG	+1 bp

We investigated off-target effects potentially caused by genome editing through the CRISPR/Cas9 system. In the genome of Japanese cultivated gentians, *AG2* is most homologous to *AG1*, with 74.4% similarity (Supplementary Figure S4). Targets 1 and 2 have eight SNPs and one SNP between the two genes, respectively. To investigate the possible off-target effects, we analyzed the sequence of *AG2* in the nine double-flowered genome-edited lines obtained. PCR products were sequenced directly, and the results showed the same sequence chromatography pattern except for line no. 6 (Supplementary Figure S5). Therefore, we subcloned line no. 6 and determined the sequence of each allele. As a result, all lines contained no mutations in *AG2* target 1, whereas one line had a heterozygous −5 bp deletion mutation in one allele of *AG2* target 2 (Table 2).

**Table 2:** Table 2. Off-target analysis of homologous sequences in *AG2* determined by Sanger sequencing analysis.

Line	Allele	Target 1 homologous sequence	In/del	Target 2 homologous sequence	In/del
WT	1	AATTCTAGGAAAAGTGGTAGAGG	WT	ACTTTCTGCAAACGCAGAAGTGG	WT
	2	AATTCTAGGAAAAGTGGTAGAGG	WT	ACTTTCTGCAAACGCAGAAGTGG	WT
No. 6	1	AATTCTAGGAAAAGTGGTAGAGG	WT	ACTTTCTGCAAACGCAGAAGTGG	WT
	2	AATTCTAGGAAAAGTGGTAGAGG	WT	ACTTTCTGCAAA-----AAGTGG	−5 bp

**Figure 3:**
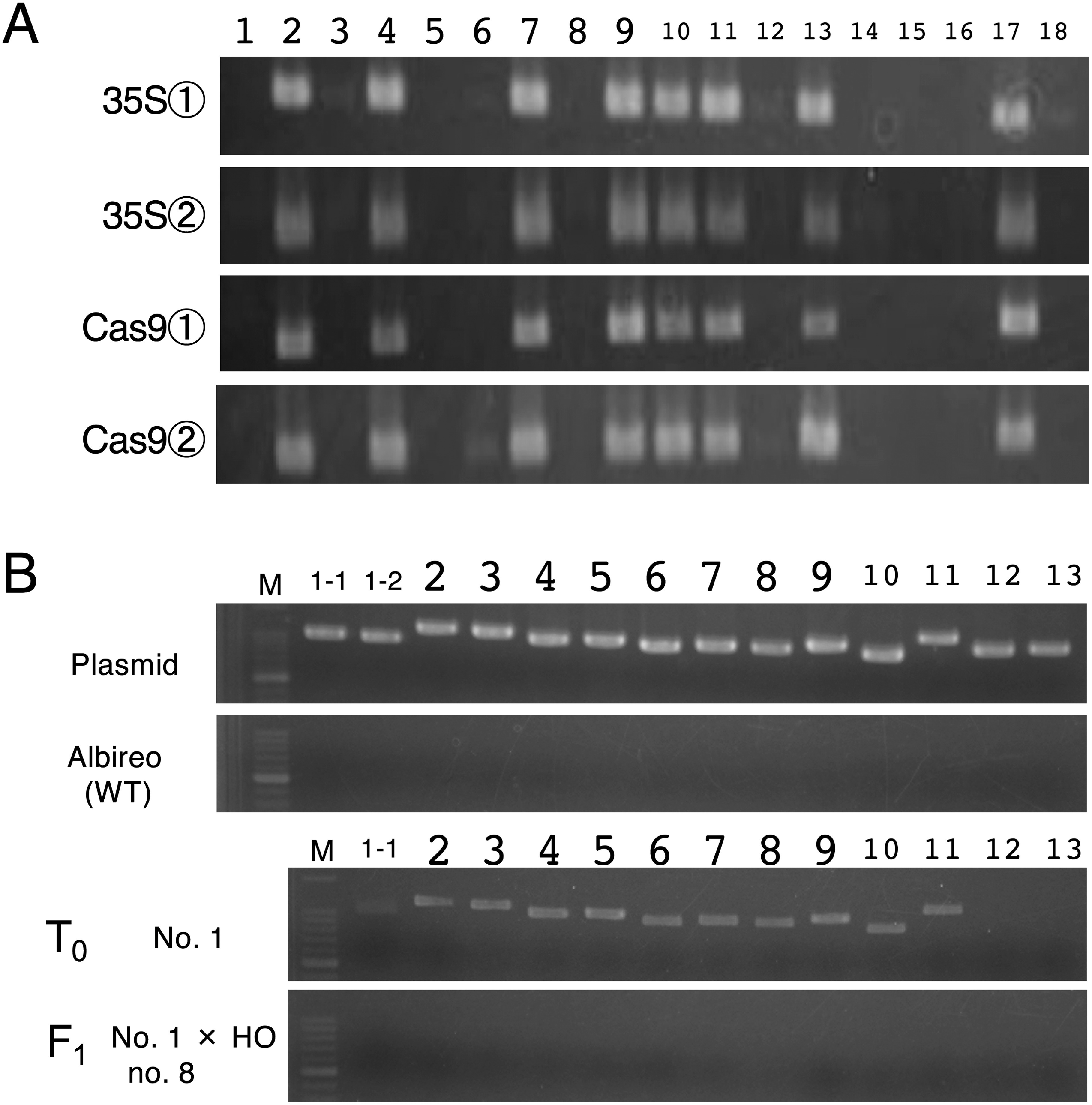
Figure 3. PCR analysis of F_1_ individuals produced by crossing the *AG1* genome-edited line and WT pollen. (A) Example of PCR analysis to screen null segregants using crude DNA extracts. Partial regions of the CaMV35S promoter and *Cas9* shown in Figure 1 were amplified in an F_1_ individual (T_0_ line no. 1×HO). F_1_ individual numbers are shown above the electrophoresis panel. (B) Confirmation of null segregants through amplification of fragments covering the entire T-DNA region. Numbers above the electrophoresis panel correspond to fragment numbers, as shown in Supplementary Figure S6 and Supplementary Table S1. pSALS-AG1 was used as plasmid DNA. Results are shown for genome-edited T_0_ line no. 1, its progeny no. 8 (T_0_ line no. 1×HO), and the WT.

To enable the practical use of genome-edited plants, it is necessary to remove the genome editing tool (here, integrated T-DNA) from their genome. In this study, genome-edited gentians were crossed with WT pollen to generate F_1_ seedlings. PCR analysis was performed on the F_1_ seedlings to detect the presence of the CaMV35S promoter and *Cas9* gene. The typical results and all results are shown in Figure 3A and Supplementary Table S2, respectively, and the PCR analysis results for all combinations are summarized in Table 3. For all crossing combinations, the scoring data of F_1_ individuals fit a 1 : 1 ratio based on a χ^2^ test (*p*>0.05), which is consistent with the expected segregation ratio assuming that the integration of T-DNA was at a single locus. In total, 46 unamplified F_1_ seedlings that did not contain the genome editing tool in their genome were considered null segregants. Two F_1_ individuals from each line were selected for further analysis, and PCR covering the entire T-DNA region was performed using their purified genomic DNA. The results showed that pSALS-AG1 plasmid DNA was amplified using all primer combinations, and T_0_ primary genome-edited lines were also amplified, except for primer pairs outside the T-DNA region (Figure 3B, Supplementary Figure S6). In contrast, when using the same primers and PCR conditions, four F_1_ individuals and the WT did not exhibit any PCR products, suggesting that T-DNA had been removed through segregation. These four F_1_ null-segregants were grown in a closed greenhouse. They all produced single flowers (Supplementary Figure S7), confirming the previous finding that double-flowered phenotype was a recessive trait (Tasaki et al. 2017).

**Table 3:** Table 3. Summary of PCR analysis of F_1_ individuals.

♀		♂	PCR		*p*
			+	−	
No. 1	×	HO	8	10	0.64
	×	unk	12	6	0.16
	×	MN	13	5	0.06
No. 14	×	HO	8	10	0.64
	×	unk	10	6	0.32
	×	MN	9	9	1
Total			60	46	0.17

*p* values were caluculated based on the expected ratio of 1 : 1 according to a χ^2^ test.

## Discussion

We successfully used the CRISPR/Cas9 system to edit the *AG1* gene in Japanese cultivated gentians and produce double-flowered plants. Among 12 herbicide-resistant plants obtained, 9 had biallelic mutations in the *AG1* target sites and exhibited the double-flowered phenotype. This outcome supports a previous study that showed *GsAG1* to be responsible for the double-flowered phenotype in *G. scabra* (Nakatsuka et al. 2015). In this natural mutant line, a retrotransposable element inserted into the sixth intron of *GsAG1* caused the same phenotype. Our biallelic mutation rate of 75% is relatively high compared with the rates obtained in previous genome editing studies targeting flower color-related genes (4.9% to 12.9%) (Tasaki et al. 2019, 2020). The use of the CmYLCV promoter to drive the guide RNA, instead of the *Arabidopsis* small RNA U6 promoter used in previous studies, may have contributed to this higher efficiency. In genome editing involving the flower senescence-related gene *EPH1L*, the biallelic mutation rate was 21.4%, and the CmYLCV promoter was used in this case (Takahashi S et al. 2022). The CmYLCV promoter, in combination with the tRNA-processing system, has been shown to be highly effective in genome editing, particularly when compared to the *Arabidopsis* small RNA U6 promoter and guide RNA tandem construct (Cermak et al. 2017). Therefore, it is probable that the high efficiency of genome editing in the present study was due to the alteration of guide RNA expression levels. Alternatively, the selected targets may have been more susceptible to mutations relative to those in previous studies (Tasaki et al. 2019, 2020). Further investigations with diverse sequences are required to fully elucidate genome editing efficiency in gentians.

A similar approach was used to create double flowers in torenia through genome editing of C-class MADS box genes (Sasaki and Ohtsubo 2020). In torenia, knockout of both *TfPLE* and *TfFAR* was necessary for double flower production, whereas, in gentians, mutation of *AG1* alone was sufficient. Although the function of gentian *AG2* remains unknown, creating double knockout lines of *AG1* and *AG2* simultaneously may provide insights into the detailed function of C-class MADS box genes in gentians. Genome editing of *AGAMOUS*-like genes also caused a late-bolting phenotype in Chinese cabbage (Shin et al. 2022). However, in our gentian *AG1*-genome-edited lines, there was no observable difference in flowering time. More detailed analysis is necessary to understand the characteristics and stability of double-flowered phenotypes under various cultivation conditions, such as in a nonclosed greenhouse or the field.

To use the genome-edited plants in nonclosed greenhouse or field settings, null segregants (transgene-free genome-edited plants) must be produced to meet the legal requirements of the Cartagena Protocol on GMO biosafety. In our study, we successfully produced null segregants from *AG1*-genome-edited lines by crossing them with WT pollen, as indicated by PCR analysis of F_1_ progeny showing an approximate 1 : 1 segregation of foreign genes (Table 3). Further detailed analysis, including coverage of the entire T-DNA region, confirmed the absence of T-DNA in four F_1_ null segregants (Figure 3B, Supplementary Figure S6). However, there is still a residual risk of partial foreign DNA fragments in the genome, which cannot be eliminated using only PCR analysis but can be analyzed using next-generation sequencing (*k*-mer detection), as demonstrated previously in rice (Itoh et al. 2020). For a more rigorous safety assessment, using more than two different methods is recommended. Therefore, we are currently conducting next-generation sequencing analysis of progeny from *AG1* genome-edited gentians to confirm null segregants. To obtain null segregants with the double-flowered phenotype, it is also necessary to produce F_2_ progeny with *AG1* biallelic mutations, and we are proceeding with crossing experiments to achieve this.

Previous studies have revealed off-target mutations in plant genome editing (Graham et al. 2020). However, the occurrence of off-target mutations in gentian genome editing has not been studied. Given the unavailability of genome sequences in Japanese cultivated gentians, we analyzed *AG2* sequences corresponding to homologous *AG1* (Supplementary Figure S4) in our *AG1*-genome-edited gentian lines. We found a heterozygous −5 bp/WT sequence in *AG2* target 2 in one of nine lines, indicating that off-targets could occur in gentian genome editing at a low frequency. However, off-targets in *AG2* target 2 occurred in only one allele, and the line showed the same double flower phenotype as that in the other eight lines. As the gentian genome size is estimated to be approximately 5 Gb (Mishiba et al. 2009), and given that the genome may contain similar sequences to *AG1* target sites that could become off-targets, further studies using next-generation sequencing are necessary to detect off-target mutations in genome-edited gentian plants.

In summary, efficient production of double-flowered lines in gentians was achieved through genome editing of the *AG1* gene using CRISPR/Cas9-mediated technology. Progeny considered to be null segregants was also successfully produced through cross-pollination. These genome-edited lines can serve as valuable genetic resources for future gentian breeding efforts.

## Abbreviations

AG: AGAMOUS
ALS: acetolactate synthase
Cas9: CRISPR-associated protein 9
CmYLCVp: Cestrum yellow leaf curling virus promoter
CRISPR: clustered regularly interspaced short palindromic repeats

## Description of Supplementary Files

Supplementary Table S1. List of primers used in this study.

Supplementary Table S2. PCR amplification of F_1_ individuals using the four primer sets shown in Figure 1.

Supplementary Figure S1. PCR analysis of crude DNA extracts of BS-resistant shoots.

Supplementary Figure S2. Sequence chromatograms of the *AG1* gene in BS-resistant lines.

Supplementary Figure S3. Photographs of flowers of other biallelic *AG1* genome-edited lines.

Supplementary Figure S4. Comparison of open reading frame sequences between *AG1* and *AG2*.

Supplementary Figure S5. Sequence chromatograms of the *AG2* gene in *AG1* biallelic genome-edited lines.

Supplementary Figure S6. PCR analysis of whole T-DNA regions in putative null segregants derived from *AG1* genome-edited lines.

Supplementary Figure S7. Photographs of flowers of putative null segregant F_1_ plants.
