# Supplementary material for: Efficient double-flowered gentian plant production using the CRISPR/Cas9 system
Source: Plant Biotechnol (Tokyo). 2023 Sep 25;40(3):229–36. doi: 10.5511/plantbiotechnology.23.0424a (PMC10901158; doi:10.5511/plantbiotechnology.23.0424a)

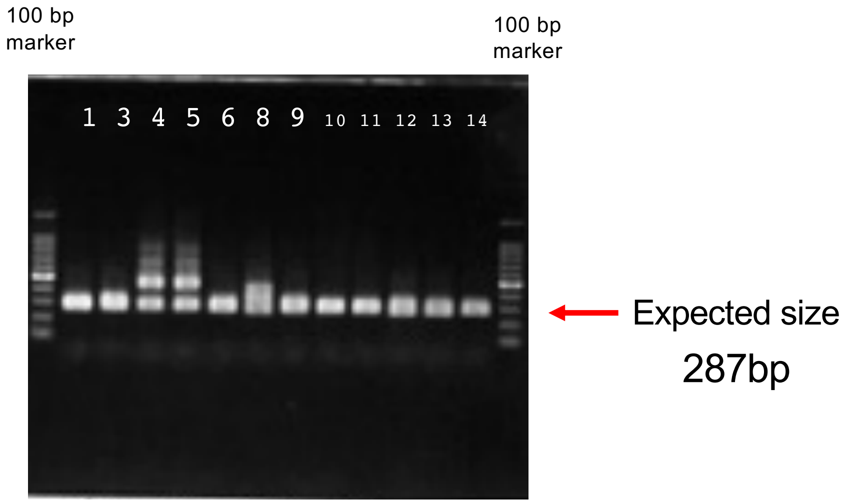

Supplementary Fig. S1

PCR analysis of crude DNA extracts of BS-resistant shoots.

Part of *AG1* was amplified using the primer pairs shown in Figure 1.

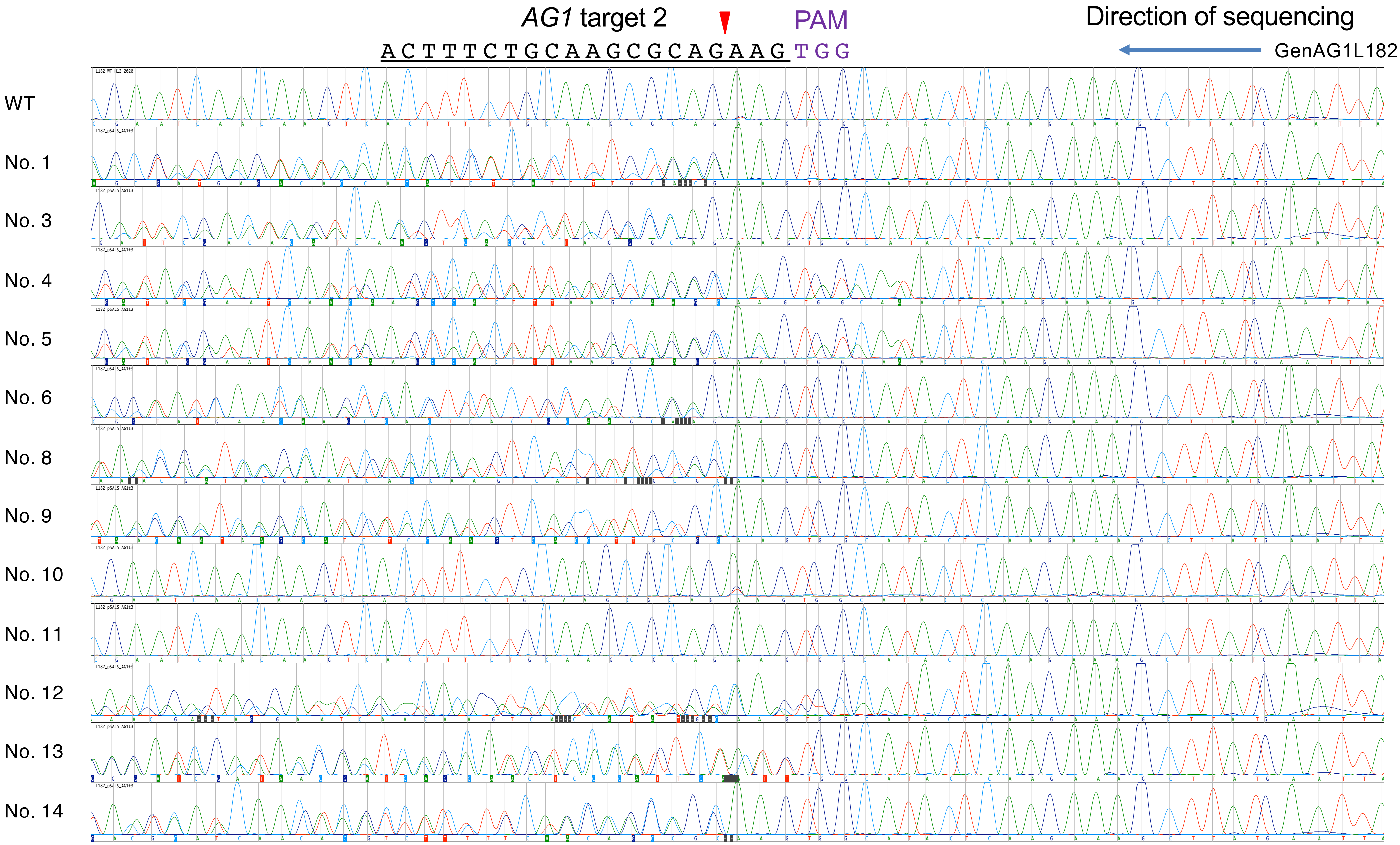

Supplementary Fig. S2 Sequence chromatograms of the *AG1* gene in BS-resistant lines.
PCR products were subjected to Sanger sequencing using the GenAG1L182 primer. The region of the sequence chromatogram including the target site is enlarged. Transgenic line numbers are shown on the left of each chromatogram. WT indicates the wild-type sequence (cv. Albireo).

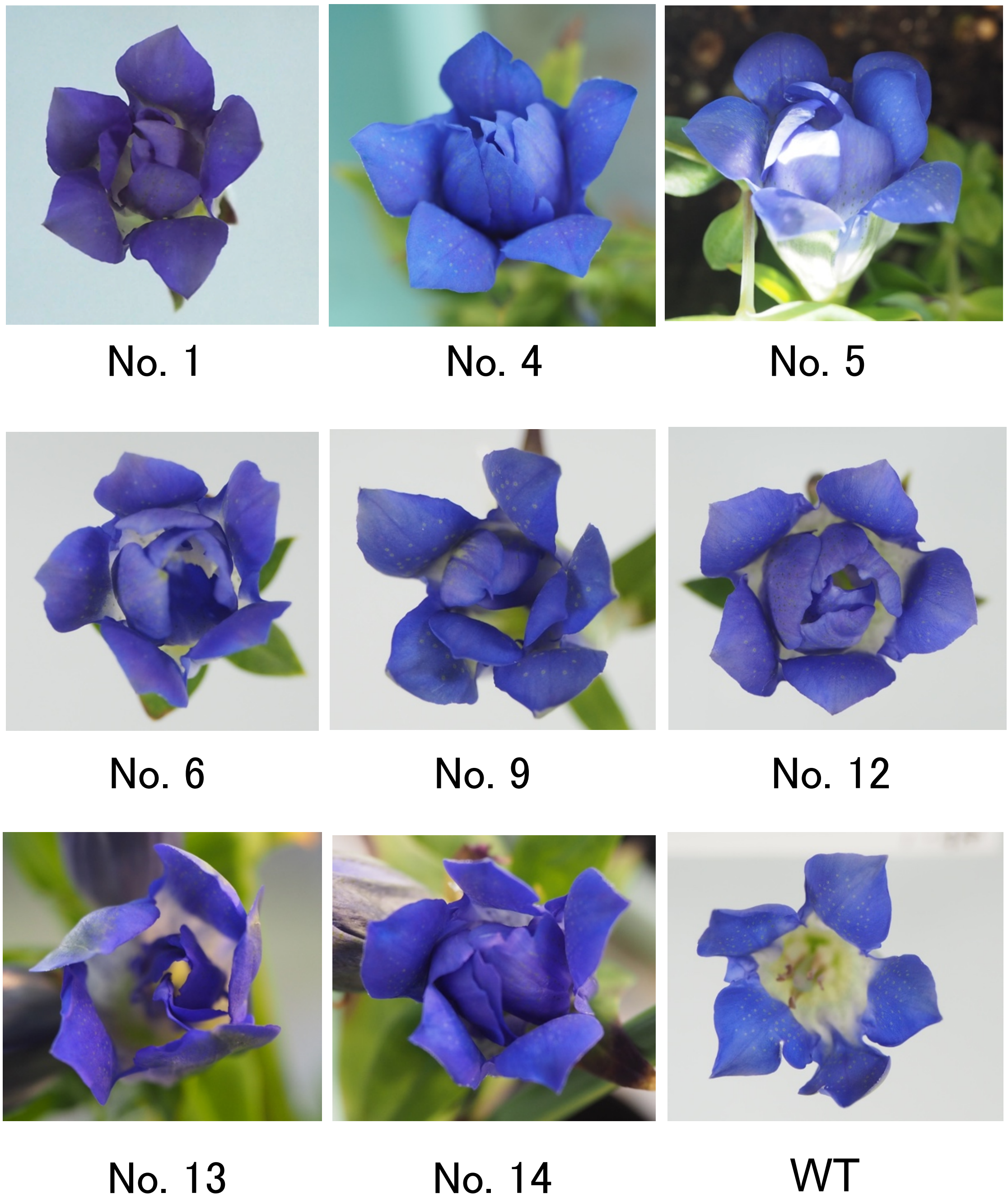

Supplementary Fig. S3

Photographs of flowers of other biallelic *AG1* genome-edited lines.

Photographs were taken 5–8 months after acclimatization. WT indicates the wild-type ‘Albireo’.

```
                                                    Target 1
AG1   1:  ATGGATTATC CTCCTAGACA AGAATTCGAC TCGTCTTCGT CGAGGAAATC AGGCAGAGGA AAGATTGAGA
          ****   **   **  *  ** **** * ** ** ***   * * ******   ** ****** **********
AG2   1:  ATGG---ATA GTCAAAATCA AGAAATTGAA TCATCTAATT CTAGGAAAAG TGGTAGAGGA AAGATTGAGA

                                                    Target 2
     71:  TCAAGAGGAT AGAAAACGCT ACGAATCAAC AAGTCACTTT CTGCAAGCGC AGAAGTGGCA TACTCAAGAA
          ******* **  ** ***   ** **   ** **** ***** ****** *** ********   **********
     68:  TCAAGAGAAT TGAGAACAAA ACCAACAGAC AAGTTACTTT CTGCAAACGC AGAAGTGGTT TACTCAAGAA

    141:  AGCTTATGAA TTATCTGTTC TTTGTGATGC TGAAGTTGCT CTTATTGTCT TCTCAACCCG TGGTCGCCTT
          ********** ******** * **********  ** ****** ******** * **** * * * **********
    138:  AGCTTATGAA TTATCTGTGC TTTGTGATGC CGAGGTTGCT CTTATTGTTT TCTCTAGCAG TGGTCGCCTT

    211:  TATGAATATG CTAGCAGCAG TGTTAGAGGA ACTATTGAAA GGTATAAGAA AGCTTGTGCT GATACCACCA
          ********** * * ** *** **  *  *** ******** * **** ***** ******** * *** **** *
    208:  TATGAATATG CCAACAACAG TGCCAAGGGA ACTATTGAGA GGTACAAGAA AGCTTGTGGT GATTCCACAA

    281:  ATTCTGGATC TATTTCCGAA GCCAACACTC AGTTTTATCA GCAACAATCC AACAAGCTGC GGAAAGATAT
           * * *****   **** *** ** ****  * **** ***** **** ** *  *** ** *   ***** * **
    278:  GTGCAGGATC AGTTTCTGAA GCAAACATCC AGTTCTATCA GCAAGAAGCA AACCAGTTAA GGAAAAACAT

    351:  CAAAGAAATT CAAAAGGCAA ACAGGAACAT GCTTGGGGAA GGAGTAGAAT CAATACAGCC AAAAG-ACTT
           * *** **  ** *   *** ****  ****  ***** *** *** *** **  ***  ***    *** *  *
    348:  AAGAGATATA CAGAGTTCAA ACAGACACAT TCTTGGAGAA GGATTAG-AT GAATTAAGCT TTAAGCAAAT

    420:  GAAGAAAATT GAAGGAAACC TTGAGAGAGC CATTGGCAAA ATCCGCACCA GAAAAAATGA ACTATTGTTT
           *****  *  *******    ****** **   ****       * ** * ** **** *****   * ***  *
    417:  CAAGAATTTG GAAGGAAGGG TTGAGAAAGG AATTGCTCGT GTTCGTAGCA GAAAGAATGA GTTGTTGGCT

    490:  GCTGAGATAG AGCTCATGCA GAAGAGAGAG ATGGAACTGC AGAATGCAAA CTTGTACCTT CGAGCTAAGA
          ******** * ******** *  ***** *** ** ***** * * ***** ** * * *****   *********
    487:  GCTGAGATTG AGCTCATGAA AAAGAGGGAG ATTGAACTAC AAAATGCCAA CCTATACCTG AGAGCTAAGA

    560:  ACTTTGATAT GGTGCAGATA GCTGAAAATG AGAGAGCCAC CACAGATCCT CATATGAACT TAATGCCAGC
               ** *   *  *    * ** ** * *    * ** **   **  * *   ** ******* * ********
    557:  TAACAGAAAA TGATC----A GCAGAGAGT- --ACAGGCAG AAC--AACAG CAAATGAACT TCATGCCAGC

    630:  ATCTGCATCT GAATATAATC ATCATCAATC CATGGCTTCA CA--TTCATT -TGATGATGT TAGAAGCTTC
          *****  *    **  ****  ** ** ***   ** ** ***  *   * ***  * * ** ** *   * ****
    618:  ATCTGATTAC CAAACTAATA ATAAT-AAT- -ATTGCCTCA GAGCCTAATT ATCAAGAGGT TCACAACTTC

    697:  ATCCCTGTCA ACCTACTCGA ACCTAATCAG CATTATTCTC GCCAGGATCC CACCGCCCTT CAACTCGTCT
          ***** ** * ** * ** **  * ******  ********     ** ***** *** ****** *** * ** *
    685:  ATCCCAGTTA ACTTTCTGGA CCATAATCAA CATTATTCCT CTCAAGATCC CACTGCCCTT CAATTTGTTT

    767:  AA
          **
    755:  AA
```

Supplementary Fig. S4
Comparison of open reading frame sequences between *AG1* and *AG2*.

cDNA sequences of *GsAG1* (accession no. LC022775) and *GsAG2* (accession no. LC022779.1) derived from G. scabra cv. Alta are aligned.

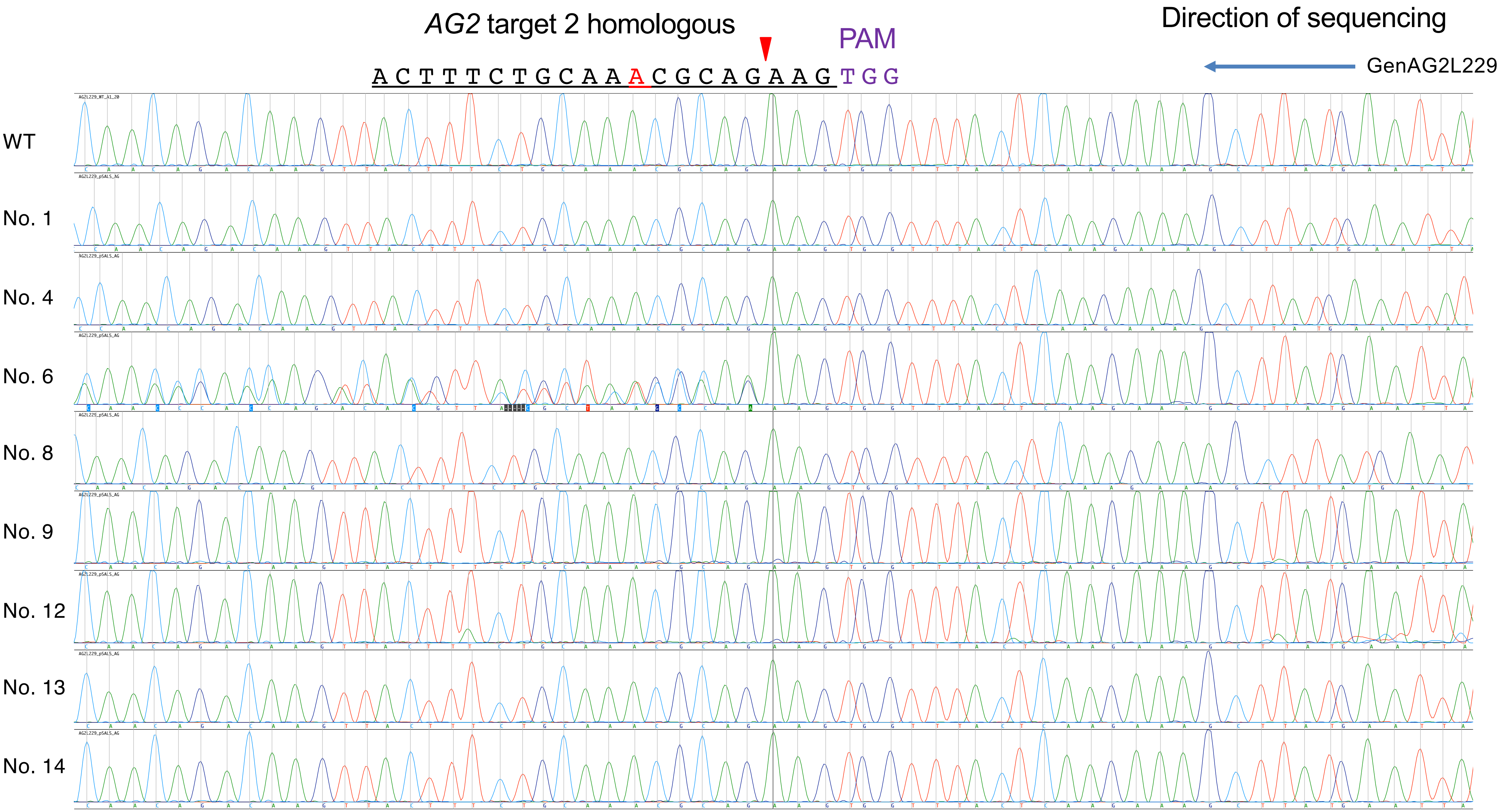

Supplementary Fig. S5. Sequence chromatograms of the *AG2* gene in *AG1* biallelic genome-edited lines. PCR products were subjected to Sanger sequencing using the GenAG2L229 primer. The region of the sequence chromatogram including the target site is enlarged. Transgenic line numbers are shown on the left of each chromatogram. WT indicates the wild-type sequence (cv. Albireo).

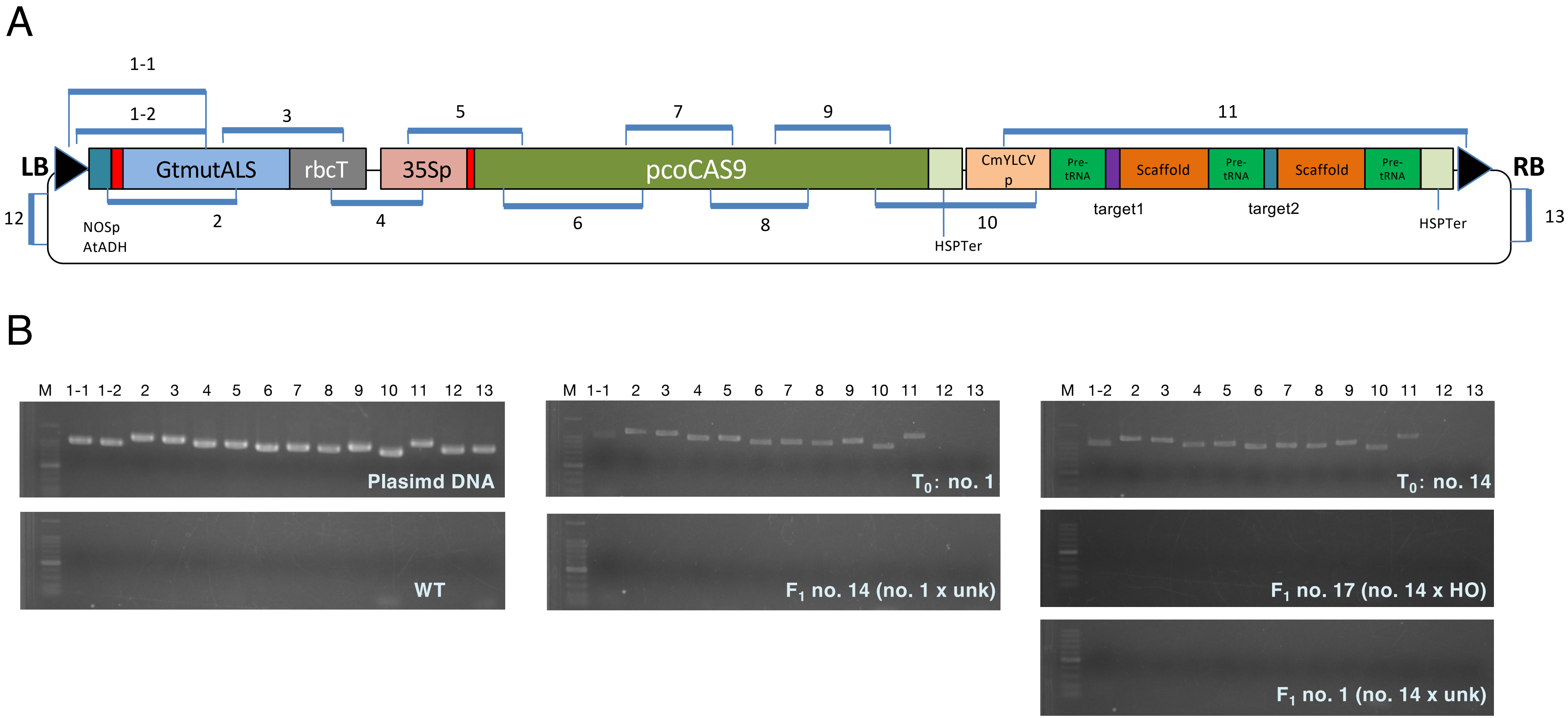

Supplementary Fig. S6 PCR analysis of whole T-DNA regions in putative null segregants derived from *AG1* genome-edited lines.

(A) Deformation map of pSALS-AG1. Amplification fragments (1-1, 1-2, 3-12) are shown. Primers and amplified fragment sizes are shown in Supplementary Table S1.
(B) PCR amplification of genome-edited T_0 lines (no. 1 and no. 14) and three F_1 lines. Amplification fragment numbers are shown above each electrophoresis panel. Plasmid DNA, pSALS-AG1, was used as the positive control. WT indicates the wild-type sequence (cv. Albireo).

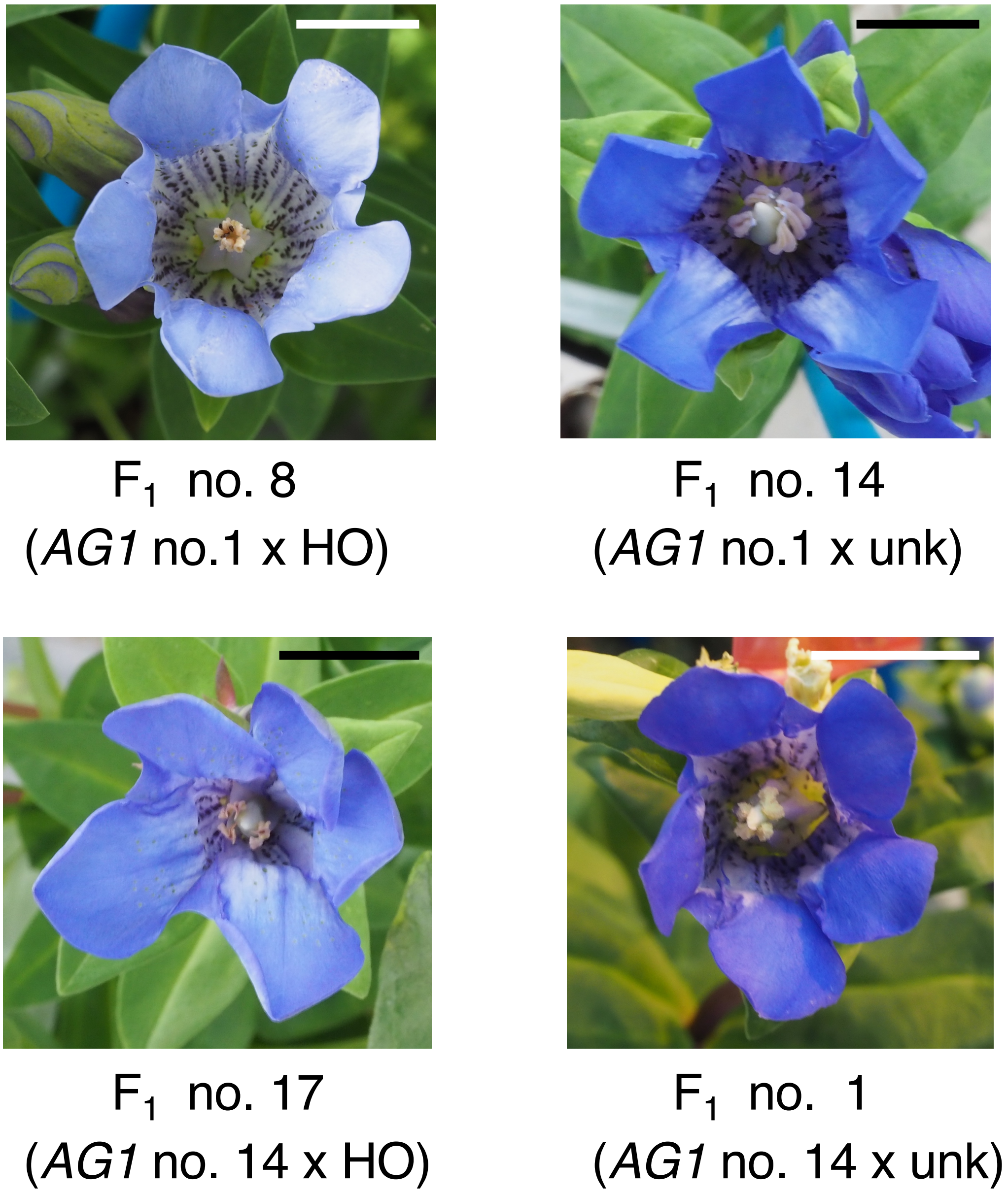

Supplementary Fig. S7

Photographs of flowers of putative null segregant F_1 plants.

All F_1 plants produced single flowers. Scale bar: 1 cm.

Table S1. List of primers used in this study

Amplification	Purpose	Forward primer	Sequence (5' to 3')	Reverse primer	Sequence (5' to 3')	Product length (bp)	Fragment no.
AG1	Analysis of mutations	AG1F(-83)	GCCTAATTAAGGCTATAATCTTTG	AG1R182	GACCACGGGTTGAGAAGACAATAA	287	
AG2		AG2F(-20)	CTCTGTTGAATTTGAAGGATATGG	AG2R229	TGTTGGCATATTCATAAAGGCGAC	243	
35Sp①	Pre-screening of null segregants	35SProF107	CTACCCGAGCAATAATCTCCAGGAAA	35SProR485	TTGCCCTTTGGTCTTCTGAGACTGTA	404	
35Sp②		35SProF305	GGTAGTTCCCACTGAATCAAAGGCCA	35SProR701	CGTCTTCTTTTTCCACGATGCTCCTC	422	
Cas9①		Cas9F1898	AAGAAAGGCTCAAGACCTACGCTCAT	Cas9R2260	TCGATCACGATGTTCTCAGGCTTATG	388	
Cas9②		Cas9F3647	CTGCTGGTGAGCTTCAAAAGGGAAAC	Cas9R4033	GTGATAGACTGATGGATGAGGGTAGC	412	
On the T-DNA	Confiramtion of null segregants	LB_F15	GTGGTGTAAACAAATTGACGCTTAG	ALS_R1036	CCTCTTTTACAATACGAGGAATGTC	1022	1-1
		LB_F39	GACAACTTAATAACACATTGCGGAC	ALS_R1036	CCTCTTTTACAATACGAGGAATGTC	998	1-2
		NOSpF236	CACTGACGTTCCATAAATTCCCCTC	ALS_R1384	CATGCATTCCCAACATCTGCAAAGA	1149	2
		ALS_F1311	AAGCACGTTGATGGGGCTGGGATCA	rbcST_R2437	ATAGTTCCTCAACTTTTAAGTCGGC	1126	3
		rbcST_F2350	ATTCAGCGTTGATTATTCTGGAACC	35SpR3389	TGGGAACTACCTTTTTAGAGACTCC	1040	4
		35SpF3310	TCCAGTATGGACGATTCAAGGCTTG	Cas9R4366	GATAGGGTGCCTCTCGTGCTTCTTA	1057	5
		Cas9F4271	TCTAACGAGATGGCTAAAGTGGATG	Cas9R5269	CAACTCTCCGAGGTGGATCTGATGA	999	6
		Cas9F5174	GAACTCCTCGTGAAGCTCAATAGAG	Cas9R6192	AGGTTAGCGATATGCTCATGGAGAC	1019	7
		Cas9F6104	CACGATGATTCTCTCACCTTTAAAG	Cas9R7106	CCTTTCCGATCTCTTGCTCAGACTT	1003	8
		Cas9F7023	TCGAGTCAGAGTTCGTGTACGGTGA	Cas9R8066	TAGACTGATGGATGAGGGTAGCATC	1044	9
		Cas9F7968	GTGCTCCTGCTGCTTTCAAGTACTT	CmYLCVpR8887	GCTCTTACCTGTTTTCGTCGTCTTG	920	10
		CmYLCVpF8750	GTTAAGGGAGCAAAATCTCAGAGA	RB_R9641	TAGTTTAAACTGAAGGCGGGAAACG	1163	11
Outside of T-DNA		LB_F16697	CGATCACCGCTTCCCTCATGATGTT	LB_R17687	CTCGGCACAAAATCACCACTCGATA	991	12
		RB_F9783	GAGTGCTTGGCATTCCGTGCGATAA	RB_R10788	CCGCTCAAAAATGGCTGGCCTACGG	1006	13

Table S2. PCR amplification of F1 individuals using the four primer sets shown in Figure 1

♀		♂	F_1	PCR
No.1	x	HO	1	-
			2	+
			3	-
			4	+
			5	-
			6	-
			7	+
			8	-
			9	+
			10	+
			11	+
			12	-
			13	+
			14	-
			15	-
			16	-
			17	+
			18	-
No.1	x	unk	1	-
			2	+
			3	+
			4	-
			5	+
			6	+
			7	+
			8	+
			9	-
			10	+
			11	+
			12	-
			13	+
			14	-
			15	+
			16	+
			17	-
			18	+
No. 14	x	HO	1	+
			2	+
			3	-
			4	-
			5	-
			6	+
			7	+
			8	-
			9	+
			10	+
			11	-
			12	-
			13	-
			14	+
			15	+
			16	-
			17	-
			18	-
No. 14	x	unk	1	-
			2	-
			3	+
			4	-
			5	-
			6	+
			7	+
			8	+
			9	+
			10	+
			11	-
			12	+
			13	died
			14	-
			15	+
			16	+
			17	+
			18	died

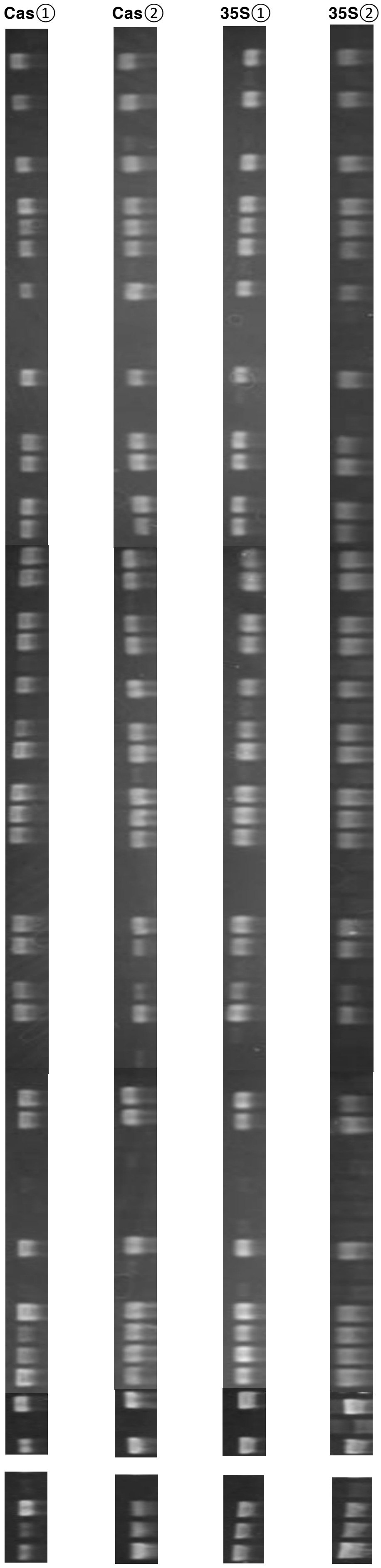

Table S2. (continued)

♀		♂	F_1	PCR
No.1	x	MN	1	+
			2	+
			3	+
			4	-
			5	+
			6	+
			7	-
			8	-
			9	+
			10	+
			11	+
			12	+
			13	+
			14	+
			15	-
			16	-
			17	+
			18	+
No.14	x	MN	1	-
			2	-
			3	-
			4	-
			5	-
			6	+
			7	-
			8	-
			9	+
			10	+
			11	+
			12	-
			13	+
			14	+
			15	+
			16	-
			17	+
			18	+

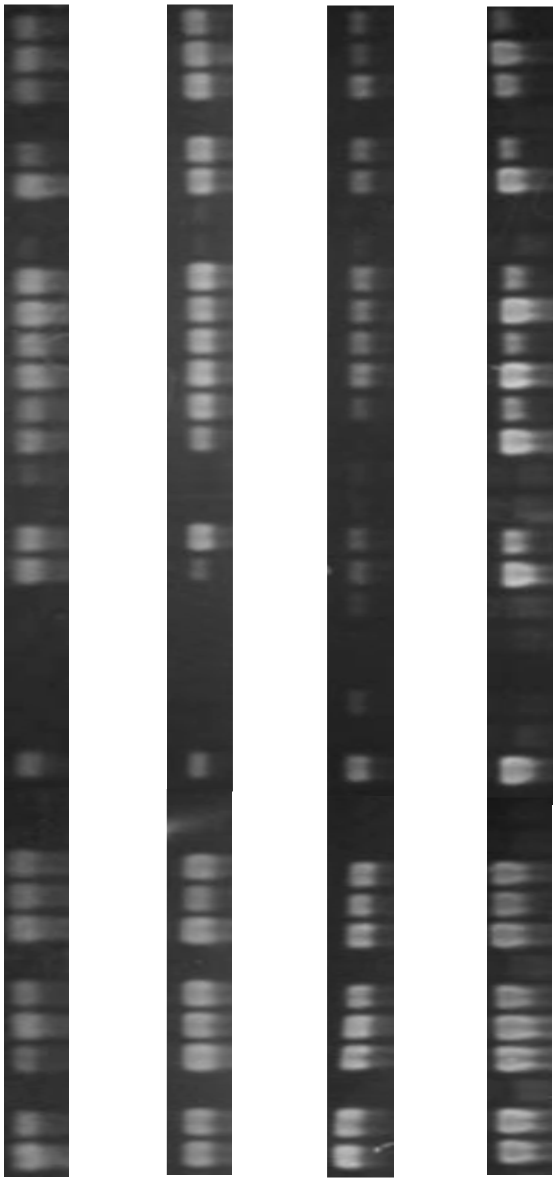

Supplement: Supplementary Data [file plantbiotechnology-40-3-23.0424a-s001.pdf]
